# Inquiry Concerning the Best Mode of Extracting the Placenta

**Published:** 1816-10

**Authors:** 


### *For the London Medical and Physical Journal.*

### *Inquiry concerning the best Mode of Extracting the Placenta;* by Interrogator.

MANUAL interferenee, in placental presentations, has been so frequently and so successfully had recourse to by eminent obstetric practitioners, that there cannot remain a doubt, in unprejudiced minds, of its occasional absolute necessity. It does not appear, however, equally decided in what manner this interference is to be directed; whether the hand is to be borne through the substance of the cake, or to be insinuated between it and the portion of uterus to which it is attached. It is in order to elucidate this point, and and to learn the opinions of some of your more experienced correspondents, that I trouble you with this communication.

The directions of authors on this head are, in general, given in such a vague and unsatisfactory manner, that the young practitioner feels puzzled how to draw any conclusions from them; indeed, if we may judge from some authors, the difference between perforating the cake and separating it from the uterus is so little, that we may act upon either plan with an equal chance of benefiting or injuring our patient. The justly-celebrated Denman gives a kind of tacit preference to separating the placenta, on the grounds that it exposes the child to less risk. If this is the result of a comparison of a number of cases, and if it can be proved that the danger to the mother is not greater than by the other method, it will at once determine us how to proceed. And though individual cases will offer such variety as will necessarily preclude the possibility of forming any rule which will serve to guide us in every instance, yet the humane and feeling practitioner will not hesitate a moment what mode to adopt when he has a chance of saving the child, without additional risk to the mother.

We will suppose a young man is suddenly called to a patient in labour. On examination, he finds the placenta presenting, attended with its usual and alarming concomitant hæmorrhage. He finds himself under the imperious necessity of immediately delivering. No time is allowed him to consult his seniors in the profession, nor can he refer to written authorities for information; and woe be to him if his mind is not previously well stored with facts and observations. The os uteri is dilating, the hæmorrhage increasing, in short, now is the time for rendering effectual assistance. The hand is gently insinuated into the os uteri, and, in effecting this, an additional portion of placenta is unavoidably separated: it still adheres, however, by a pretty broad circumference; and the question naturally arises whether it is adviseable to go on with the separation on one side of the os uteri, or to introduce the hand through the centre of the cake. As hæmorrhage is the only cause of danger to the mother, that method in which the loss of blood is the least will, of course, be the most safe; and, as the quantity of blood lost must, in a great measure, depend upon the time occupied in delivery, that plan by which it can be executed in the least time will, *cæteris paribus*, be the least liable to risk.

The thickness of the placenta in the centre, and the increased diameter of its blood-vessels, are strong objections to perforation; yet, the extent of surface of placenta which may still adhere on every side, after the os uteri is sufficiently dilated to admit the introduction of the hand, seems to render this mode equally objectionable. It is probable, however, that one of these methods may be, in some degree, preferable to the other; and, in however small a degree it may be, we ought to know and act upon it. Cases precisely in point, fortunately, seldom occur—my practice does not furnish me with a single instance. They may, however, occur, and we ought to be prepared with the best plan of remedying every difficult case. I, therefore, propose, as a question to the veterans of the profession, "Whether is it better to bore through the centre of the cake, or separate the placenta from the uterus, in cases where, as far as can be ascertained, the centre of the placenta is placed *directly* over the os uteri, and no part of the placenta is detached from its adhesions, but to the extent to which the os uteri is dilated?"

If the medical world, in general, is as unsettled on this head as my medical acquaintance, who, by the bye, have nearly as many opinions as heads, the solution of this question will be no small benefit. To young men, in particular, a clear and decided rule, where it can be given, is an unspeakable satisfaction. It destroys their doubts and uncertainties,—makes them act with that decision and promptitude which can alone, in many cases, save their patients,—and gives them a confidence in their own powers, than which nothing is more soothing and consolatory. To the presumptuous, and often ignorant youth, who fancies himself all-skilful and all-wise, I shall appear to have written unnecessarily; but the modest and diffident young man will know how to appreciate the value of my communication: if it serves to remove the doubts of one of these, I shall be amply satisfied. The bed-side of a woman in labour is a dreadful scene for hesitation, doubt, or uncertainty!

*Rochdale; Sept.* 5, 1816.

